# 

**Published:** 1860-06

**Authors:** 


### A CALL.

THERE will be a meeting of the Mississippi Valley Association of Dental Surgeons on the second Tuesday of June, at 7½ o'clock, P. M., at the office of Dr. Jas. Richardson, No. 271 Walnut street, in this city, for the purpose of appointing delegates to the National Association, to meet at Washington City in July next. It is desirable that there should be a full attendance.

GEO. WATT, Sec'y. W. H. ATKINSON, Pres't.

### CLOSE OF VOL. XIII.

This No. closes the volume, and with it expires the term of subscription paid for by nearly all our subscribers. We trust they have found it sufficiently interesting, instructive, pleasing and useful to induce them to renew their subscription without delay, and use their influence with others to become subscribers.

As the terms of the Register are

STRICTLY CASH IN ADVANCE, and as we regulate the size of the edition by the list of subscribers, it is important that names shall be sent in early, say by the 15th of June.

### EXTRA INDUCEMENTS.

As there are still many who do not take the Register, I have concluded to give an opportunity of trial to all, by issuing A VOLUME IN SIX MONTHS, from July to January, the price of which will be only ONE DOLLAR FOR THE VOLUME.

This is done partly to bring the volume so as to run current with the year, beginning with the January and ending with the December No., and partly as an inducement to those who have not been readers to try it at a trifling expense, trusting that it will soon become so welcome a visitor that they will become permanent subscribers at the regular price.

### UNPAID SUBSCRIPTIONS OF VOLS. X., XI. AND XII.

In becoming publisher and proprietor of the Dental Register, I purchased with it all its book accounts and back claims. This I did in good faith, under the assurance of the former publishers that the claims were all correct. I found, however, that the proper attention required to make collections greatly increased my already heavy correspondence, and taxed my personal time and attention beyond my abilities, whilst I was compelled to refer to Dr. Taft for all explanations, and as the former publisher, Dr. Taft, was bound in his contract to see all claims collected, as far as could be legally, I have referred the whole matter *back again to him.* I have, therefore, nothing more to do in regard to the old accounts. Those who are still indebted to the Register, can remit to Dr. J. Taft, 56 West Fourth street, or to his Attorney, John H. Piatt, Vine st., above Fourth, Cincinnati, O.

### BUSINESS NOTICES.

"Once more upon the waters! Yet once more!
And the waves bound beneath me as a steed
That knows his rider. Welcome to their roar!
Swift be their guidance wheresoe'er it leads!!"

### VULCANITE! CORALITE!! AMBER BASE, &c.!!!

AM. H. R. CO.! *vs.* ROBERTS!! DIFFENBACK, ETC.!!!

Agreeable to a promise in the January and March Nos. of the Register, I appear again before you. I have sometimes half regretted making the promise, because so few of the profession seem to appreciate the motives which have induced me to assume the expense and labor of my position. But feeling it to be a duty, I can not resist the impulse even though the task be thankless and the benefits received with ingratitude.

In the Jan. No. I intimated some doubts as to the manner and means by which Goodyear obtained an injunction against Roberts; and in subsequent articles I have asked the question, Why the agents of the American Hard Rubber Co. have not in their numerous circulars stated the case fully as it appears on the Court Record? This they have failed to do.

On page 8 of a new quarterly called the "Vulcanite," issued under the auspices of the Am. Hard Rubber Co., and edited by Dr. B. W. Franklin, the following language is used:

"Another suit was immediately begun against him (Roberts), which he made preparation to defend, by employing able counsel and entering an appearance at Court; but finding on a thorough examination of the case, that a defense would be of no avail, he failed to appear at the time of trial, and a verdict was rendered against him, with costs; and he has since paid damages to Mr. Goodyear. The decision of the Court in this case is as follows:"

The language of the decision I need not repeat, as it was distributed to the profession in the form of circulars and must still be GREEN in their memories.

This would indeed seem to be conclusive, were it not for the suspicious circumstances surrounding the case; but when we come to look at the matter more closely, doubts—unpleasant, incredible and discreditable—will arise.

In the latter part of July of last year, when in New York, I had several conversations with Roberts and on all occasions he expressed himself determined to pursue the case to the last point of law, with every confidence in his success. At the meeting of the American Dental Convention at Niagara, he exhibited the same sanguine determination and expressed it in even more positive terms. Yet in two short months after the convention, we find an injunction entered against him because *he failed to appear* at the time of trial. Why did he fail to appear? Has any person yet been able to get him to state what induced him not to appear? I myself addressed him several times but could extort no reply. At last, however, he said he would explain when he had leisure.—I presume he has not yet been at *leisure!* The A. H. R. Co's. agent states it was because he found a defense would be of no avail!

Ah ha! But what was it that so suddenly unfixed his defiant resolution and at the same time initiated such a cordial friendship between himself and the A. H. R. Co. that they at once became interested in the sale of "ROBERTS' Vulcanizers" and proceed to advertize them in the veritable circular in which they announced the "Question Settled?" Does it look reasonable in parties who obtained judgment and injunction against another, to wheel squarely around on the left heel and from persecuting and prosecuting go to patronizing, yea, even advertize the sale of his goods which before they had not done. Hold up the veil! does not the idle wind rend it. Hold the pretext at arms length; how does it look? Meanwhile Roberts himself goes on in the undisturbed prosecution of his business as happy as the day is long. Never before so happy and prosperous—manufacturing Vulcanizers, practicing Dentistry, etc. What has sealed the lips of him whose nimble tongue was ever ready to rail against the A. H. R. Co.? Shall we never hear his gentle voice again? Is it to be forever hushed in GOLDEN silence?

"Speech is silvern, silence is golden;
Speech is human, silence is divine."

Both parties seem to have *fallen* into sympathy with the Irishman who declared that he "*never* really *loved* a man until after he had had a *fight* with him."

I can not see what Roberts had to gain by ceasing to defend the case, nor do I believe that he would have been beaten, but even if he should, it would cost only a trifling few hundred dollars more for witness and court fees, and what is that in a contest on *principle*—it would at least have proven to his brethren that he was in *earnest.* The eyes of the entire profession were upon him, and the most of them had confidently hoped that in Roberts they had a

Champion who when he "put his hand to the plough would not turn back." "Alas! how frail are human calculations!" An honorable member of a liberal profession should be willing on all proper occasions to sacrifice self interest for the protection of that profession and the vindication of a professional principle, but

"He who fights and runs away,
May live to fight another day."

I have been accused of asserting that the case in point was COMPROMISED, and that Roberts was paid for not appearing on trial. I have no recollection of ever making such a statements and don't believe I ever did, because I did not *know* that such was the case, although I do think it looks suspicious, and I have a letter from a gentleman in New York in which it is stated that Roberts was induced, and then agreed not to appear in defense. How reliable this is, I am unable to say, but will take the liberty to present to the profession the following *paper*, taken from the *Records* of the

U. S. Circuit Court, Southern District of New York.

GOODYEAR'S ADMINISTRATOR *vs.* ROBERTS.—*In Equity.*

Please take notice that the above named defendant will make no further defense in the above action, and he hereby consents that a deeree therein may be entered against him.

(Signed,) S. D. LAW, *Atty. for Defendant.*

*New York, Sept.* 2, 1859

To G. D. SARGENT, ESQ., *Sol. for Complainant*, 30 *Nassau st.*

*Oct.* 3*d*, 1859. Ordered that a decree be entered in pursuance of the above consent.

(Signed,) S. NELSON, (Judge of the above Court,)

This CONSENT means something or nothing. Now there was no necessity for this "consent" even in the worst strait, because Roberts' failure to appear would have insured judgment against him by default. But it would not look well IN PRINT that injunction and judgment were obtained by *default*. The "*consent*" is an *appearance* whereby the case is dismissed by defendant. He must have been *desperately anxious to be beaten*, not to be able to wait for the due course of law, and so to run forward as it were to beat himself!!

The profession can now judge what reliance there is to be placed in the printed circulars of the A. H. R. Co., or the verbal statements of their local or traveling agents. Their statements all have the appearance of truth; their language is carefully and well chosen; their sentences are cunningly devised; as they state their case, it is true. But, a truth half told often becomes a falsehood. Whether it is so in this case or not the profession must judge for themselves.

In the January "Business Notices" I merely hinted my suspicions. Lo and behold the "shaking of dry bones" that follows! It was immediately announced (I presume from head quarters) to all local and traveling agents, that an article would be written and published giving me fits! Good No. 1! That papers and evidence were all prepared to enter suit against me instanter (and of course ANNIHILATE me.) Good No. 2!—for what?

That Toland has abandoned his position and has ceased to sell machines, materials, or to vulcanize.—Good No. 3. This would be the best joke of all for the A. H. R. Co, and its agents. But this I positively contradict; and any one who doubts me need only send a piece to be vulcanized, or order a machine and materials, with cash enclosed, and see if he don't receive it by next mail or Express.

Having an extraordinary confidence in the veracity of the A. H. R. Co. and all its attachees, I became greatly alarmed and made some inquiry of my Medical friends in regard to the matter. They persist in assuring me that no symptoms of fits are perceptible; and yet as I can not doubt the assertions of the A. H. R. Co., I must believe that I *have fits!!!*

In the March No. of "Business Notices" I gave a general survey of the subject,—which, with the exception of a few typographical errors which crept in, I consider correct doctrine, and by that I am willing to abide.

The kinds, qualities and prices of some materials have changed, but that is an every day occurrence.

When that article was written, I had the assurance of Dr. Diffenback, through his agent Chas. Wehle, Esq., that they were courting a legal investigation, and would defend any person who might sell, manufacture or use their materials; but like the rest they have melted as snow beneath a summer's sun,

I learned sometime ago, from good authority, that Dr. Diffenback had abandoned his position. Yet I had such confidence in him that I could not believe it. More recently facts have been developed and there is no longer any doubt but that Dr. Diffenback has procured an office right and signed a paper to the effect that his Amber Base can be used, only subject to the Goodyear patents. What a lame conclusion to so stout a resolve! Does such a cowardly and humiliating course require any comment?

But what follows hereupon? In the "Vulcanite," a new quarterly published by the Am. H. R. Co., May No., is a card over the signature of Dr. Diffenback, in which occurs this language: "In offering it to the profession, I have thought and do honestly think, that the preparing and selling of my "Amber Base" is no infringement upon the Goodyear patents, and which fact has been ACKNOWLEDGED by them and their agents;" and then: "but on learning, however, that the American Hard Rubber Company, owners of the Goodyear patents, were preparing to commence suit against me for infringement upon their rights, I thought it judicious to take legal counsel in the matter; and after a full and careful examination of all my own and the Goodyear patents, I am advised that my process of vulcanization, as well as the moulds used by me in that process, are covered by the Goodyear patents."—A flat contradiction, and in strange contrast to that, is the following paragraph taken from a circular issued by Dr. Diffenback but a few months ago, which reads as follows:

"This patent secures to me the right of using or making the Amber compound in its rigid and hard state.

"The profession will perceive by these extracts that I am not dependant on any other patent whatever for making and using the Amber base; and I am prepared to protect, defend, and save harmless, all purchasers of licenses, rights, or of the Amber composition, by reason of their purchasing, selling, mixing, hardening, coloring, wearing or otherwise using the same; and I will add, that I have frequently invited the licensees of competing patents to a judicial investigation of the merits of our respective patents—but they have hitherto declined. The Dental profession has been frequently threatened, bullied, humbugged and blackmailed by the agents for Vulcanite, and I am determined to do my share in stopping all further aggression on their part. Hence, I call on my professional brethren to aid me in this undertaking, by spurning their threats, by refusing to pay them blackmail, and by discontinuing the use of their obnoxious compound for dental purposes."

Oh! Consistency, thou art a Jewel!!

Why is it that Dr. D. deems it judicious to take legal counsel now? He has never been without it, his agent to whom all his business and correspondence was entrusted gives his card as

C. WEHLE,
Engineer, Architect and
PATENT AGENT.

Such *engineering* as this it will be difficult to reconcile with known laws.

And now Dr. Diffenback and the H. R. Co. pull together as well as if they had been pulling one way all their business lives; and the H. R. Co. takes Dr. Diffenback with all his "acknowledged" rights and folds them as nicely under its wings as a hen does her chickens. Thus, one would think the Dr. felt secure as well as comfortable, and would forever nestle in the rich down of this imaginary hen. But no! the Dr., as we have been advised by a no less respectable authority than his responsible agent, Mr. Wehle, has "jumped from under with large speed." Mr. Wehle writes us that Dr. Diffenback has left for Europe on a PLEASURE EXCURSION to be gone three or four months; and moreover, that he stood not at all upon the order of his going, but went at once. He said nothing at all to his agent and attorney, Mr. Wehle; and this strange and unwarrantable course Mr. Wehle condemns in no measured terms, as he himself expressed it, "I would have declined any participation in Dr. D.'s Amber business had I forseen his imprudent conduct." But of course as to how he settles Dr. D.'s private business I have nothing to do. I simply mention the fact. But may not the question be asked: Why, after all his difficulties had been peaceably and amicably settled, did Dr. Diffenback leave the country, and in such hot haste?

And now I come to myself: I have received intimations from various parties; agents of the Am. H. R. Co., and others; and I have been directly threatened, too, with prosecution. Strenuous efforts have been made to intimidate me; and that in a manner to affect my business. The annoyance, to say the least, has become too aggravating to be quietly tolerated much longer, and if this business is not stopped, and that suddenly, I may myself be tempted to try what virtue there is in law.

Must I be bullied, browbeaten, threatened and misrepresented by these hungry wolves, and have no redress?

Some friends have asked whether I have well *weighed* the *expense*. Have I not Franklin's treatise on the strength of metals and other materials which are ascertained by *weight?* Then why should I be at any loss? But cost what it may, I know my rights and knowing, dare maintain them.

After all the threats of this would be monopoly who boast of having $150,000 laid aside especially for litigation, what does it amount to. Each of the parties, with whom they have had any signs of litigation, viz: both Roberts and Diffenback, appear to come off the *g iner*, at least they both seem to be *more prosperous* than before. The one in business, the other in pleasure.

Like others of their kind they are always ready to commence a suit whenever they are sure of a favorable termination as was no doubt the intention when their agent, Dr. G. F. Foote, presented to me a paper for signature which reads as follows :

(*Original.*)

" *To the American Hard Rubber Co , or whoever it may concern, Greeting:*

Take notice that I, the undersigned, a citizen of Cincinnati, State of Ohio, do hereby acknowledge and state, of my own free will and accord, for the benefit of all concerned, and to be used as evidence in any suit pending or that may hereafter be commenced against me by the American Hard Rubber Co. or their agents, or by any other party, viz: That I have manufactured and caused to be manufactured, vulcanized rubber plates for Dental purposes to the amount of fifty pieces within the past year. Of these about — were made of the gum known as the Amber Base. about — pieces of the gum known as Wheat's gum, and the balance from that known as the American Hard Rubber Co.'s gum. These I have made and sold for my own individual benefit, receiving compensation therefor. And furthermore, I am making and shall continue to make and sell the same."

A friend has just remarked that "the Dr. never opens his mouth but he puts his foot in it."

To sign such a paper would be to confess judgment, and of course if I would furnish all the proof and then *confess judgment* thereon, they might with as perfect *security* enter suit against me as they have against others.

As I stated in the March No. of the Register, a patent is in itself presumptive evidence of its validity. This fact applies equally to all patents.

The requisites of a patent are originality, novelty and utility.

The list of patents issued from the U. S. Patent Office for improvements in Vulcanizing India Rubber and other gums, as recorded in the Patent Office, numbers some 20 or 25 different patents issued since 1843. In Europe there have probably been issued as many more. Will any one pretend to say that *one* of these is *valid* (and swallows up all the rest,) and that all others are worthless? Is it to be presumed that the U. S. Commissioner of Patents is a fool, that he whose duty it is to be informed, and having all the facilities of information, by reference to Court Records and consultation with the Attorney General of the U. S., and especially on a subject which has been constantly in discussion and litigation—that he should issue patent after patent (many of them *prior* to those of Nelson Goodyear) and yet all invalid except his alone—it certainly seems that " a *little* learning is a dangerous thing."

The Goodyear patents state in their claim that the said manufacture or artificial substance is produced by the admixture of India Rubber or other vulcanizable gums and sulphur, in the proportions of one pound of the gum to about from four ounces to a pound of sulphur, whether alone or with other substances and then subjected to a high degree of heat which should not be less than 260 to 270 degrees Fahrenheit scale during a period of six or more hours, or until the required degree of hardness has been obtained."

" And although much latitude may be taken in the proportional quantity of sulphur, a proportion much *less than four ounces* to the pound of caoutchouc wil lutterly fail to produce the new substance or manufacture herein above described."

Will any one have the audacity to say that this covers all vulcanization? The patent states no such thing, but only a compound of sulphur and india rubber in the proportions of from four ounces to a ponnd of sulphur with a pound of gum. No one can patent a *principle* and no one can obtain a patent without a principle. No one can patent sulphur and no one can patent India Rubber or other vulcanizable gums, but they can patent the combination in *certain proportions* to produce a certain result; this is all, they can no more patent a result than a material.

Will any of the licensees of the A. H. R. Co. be kind enough to inform me " scientifically" *what* they use in the formation of vulcanite plates? Is there any one of them who knows the precise composition of the article they purchase or use. Or have they paid their $100 simply for the *privilege of purchasing* the A. H. R. Co.'s (NOSTRUM) *gum* at $3 per pound, with its *fine texture!* and beautiful *life-like color!!* It is barely possible that they have bought a "pig in a poke." Dentistry is known as a " liberal profession." When they exhibit themselves in this wise, I think they should be characterized as a "*prodigal* profession," and I expect to see the day when like the "*prodigal son*" they will return to the teachings of their parent in true contrition, acknowledging, " I am not worthy to be called thy servant, much less to be accounted thy child."

The agents of the American Hard Rubber Co. acknowledge that they can not claim *broadly* the *manufacture* of *material*, but then "you can not vulcanize without a right." Why not? In the patent *specification* of Nelson Goodyear's *Re-issue*, this remarkable language occurs, in *contrast* to the pretentious assertions of the agents of the A. H. R. Co.: "I do not wish to be understood as making claim broadly to a manufacture or substance produced by the admixture of Caoutchouc and Sulphur nor as making claim broadly to a manufacture or substanc by subjecting the compound to a high degree of artificial heat as prior to the invention of the said Nelson Goodyear. Caoutchouc and sulphur had been compounded, and such compound alone as well as with other ingredients had been subjected to a high degree of artificial heat."

To sum up the whole matter the Goodyear patents cover the munufacture of a hard and inflexible substance composed of india rubber and other vulcanizable gum, in the proportion of a pound of gum to from four ounces to a pound of sulphur, and NOTHING MORE.

I have further to remark upon the mode of doing business. Unable to rest upon the merits of their article and their claim, their agents, as I am informed, have made all sorts of *ungentlemanly* remarks and insinuations misrepresenting my position and course entirely; the game seems to have been *any thing* to effect a *sale*. I have pursued the even tenor of my way openly and aboveboard and shall continue so to do, if my position and reputation are not sufficiently established to shield me from *unprincipled assaults* I will even have to suffer, but as I hope in the eternal principles of truth and justice, I do not fear the final result.

Dr. Franklin in the January No. of the N. Y. Dental Journal, says: "How any man having any regard for truth from facts that are within his own knowledge, can charge the A. H. R. Co. with any particular 'urgency manifested to sell rights,' we can not tell." I think there are a large number of dentists in the west who could tell, even if Dr. F. can not. The agents (or at least one of them) have been so anxious that they have offered to loan machines, flasks, &c., to persons who would use them for any desired length of time, so that when they became favorably impressed he would have the first chance to sell a right. This may be a legitimate course of business, but it is not my mode. However, all such matters depend upon "*the way in which a man is brought up.*"

That I may not be misunderstood I repeat again: The patents of Nelson Goodyear deceased gives the right to the combination of India Rubber and other known vulcanizable gums with Sulphur in any proportion within the limits of one pound of gum with from four ounces to a pound of Sulphur, and to VULCANIZE THE SAME, there it stops. A combination of India Rubber or other vulcanizable gum, containing a *much less quantity of sulphur than four ounces* to one pound of gum, or *much more* than *one pound of sulphur* to a pound of gum, s outside of the letter and spirit of their claim and is NO *infringement*.

In assuming to control that which their patent does *not* cover, let them beware lest they jeopardise that to which they have an *apparent* claim, since a thorough sifting might possibly discover defects in their recorded claims, which they might find it difficult to cover up.

I am not aware that I have infringed the rights of the A. H. R. Co. or any other legal patent, but if they think otherwise, and think it their interests, and think they can sustain an action against me, why not proceed at once? why these repeated threats? I am a peaceable and peace-loving citizen but would much prefer to have the suit commenced than this annoyance of attempted intimidation.

Some have said, "Toland is not doing right in opposing this thing." I have only opposed it so far as it has undertaken to assume rights which the recorded specifications do not grant to them; but because others have not the moral courage to vindicate a principle against a wealthy monopoly—they have no right to censure me. Time will determine which is right. I occupy now the same position as heretofore, and on principle will defend it. Whether I will have any thing more to say on this subject, will depend on circumstances; I have no love for controversies of this kind, but must do my duty, hoping however not to have occasion soon again to trespass upon your time and patience.

I am prepared, as heretofore, to furnish the most approved Vulcanizing aparatus, Flasks, Scrapers, Thermometers and all kinds of Vulcanite materials, with complete instructions, (printed or oral.)

JNO. T. TOLAND,
38 *West 4th st., Cincinnati, O.*

POLISHED TEETH.—I am just n receipt of a fine assortment of Orum & Armstrong's polished Teeth, which for natural, life-like appearance, far exceed even their heretofore beautiful productions; the polishing takes off that glossy glare which so readily leads to the detection of artificial teeth in the mouth, especially at night under a strong artificial light. The price is the same as for the ordinary kinds, viz : 20 cents for Gum Teeth, 10 cents for plain.

JONES AND WHITE'S PREMIUM TEETH.—Of these it is needless to speak, as every Dentist is familiar with their merits. Suffice it that I have at all times a large and carefully selected stock and receive from $2,000 to $3,000 worth per month direct from the manufactory, and the proprietors inform us they can not make them with sufficient rapidity to supply the demand. Teeth especially for *vulcanite work* in Sections, Single Gum, and plain in great variety.

OSTEOPLASTIC.—Prof. Pearson is still in the field and pushing along; the demand for his article is truly astonishing and rapidly increasing, whilst the testimony is almost universally satisfactory. See advertisement.

OS-ARTIFICIAL.—This article is having a large run and gives great satisfaction, and for temporary fillings will soon supplant the use of Amalgams, Hill's Stoppings, &c.

FRENCH INSTRUMENTS.—The rapidity with which these have gained favor is unprecedented, however, not at all surprising, the quality of the instrument considered. They cut like a razor and the *temper perfect;* the only difficulty is to keep sufficient supply, which, however, I am making every exertion to do.

NEW ILLUSTRATED CATALOGUE, containing 70 pages and nearly 200 engravings. Some errors and imperfeccions have crept in, but those who know me will need no assurance that orders will be filled promptly, accurately and at correct prices. Copies will be sent FREE on application.

NEW BLOWPIPE, with ball to open in the center so as to admit a small piece of sponge which absorbs and retains the moisture of the breath. Illustrated on page 38 of catalogue, price $2.00.

CRYSTAL GOLD FOIL.—My arrangements are now complete to supply this superior article fresh from the manufacturers in any quantity. The increased demand is great indeed, but an article of real merit will always meet with appreciation, sooner or later.

TOLAND'S GOLD FOIL.—I have been delayed somewhat in bringing this article before the profession, but in a few weeks will have an abundant supply to fill all orders. It is not proper to say much in advance, but I feel confident that this article will be of such uniformly superior quality, that the profession may at all times depend upon it for correct results.

GOLD FOIL, of Jones & White's, Dunlevy's, Leslie's, Chas. Abbey & Sons', and Morgans, and also A. J. Watt's Crystal Gold, always on hand, I keep only the *best* of everything.

FORCEPS AND OTHER INSTRUMENTS.—The beauty and superiority of our instruments have created a demand which I have found difficult to supply. I have now made arrangements which, I think, without doubt will enable me to supply the profession promptly with articles of superior merit.

IMPROVED VULCANITE FLASKS, with long guide pins. Price 50 cents each.

LARGE CLAMP, to screw fast to the work-bench. To be used for bringing Flasks together. Price $2.00.

PREPARED PARAFFINE, according to the mode of Dr. R. Peckover. This bids fair to be a valuable acquisition, in the place of wax for impressions. It is clear and beautiful and takes a sharp, accurate *impression*. For the vulcanite work to be used for the model plate it is far superior to Gutta percha or "impression gum," as it is much more easily handled and is neat and clean and can be used over and over again as often as is desired. It is just the thing, avoiding all warpage or springing of the model as is the case with Gutta percha.

In cakes for taking impressions, &c., .................................. 75 per pound.
In sheets rolled out thin to be used for vulcanite work, ............. $1 50 " "

MEMPHIS DENTAL DEPOT.—After frequent solicitations from numerous Southern friends, I have opened a Depot in Memphis, and I am gratified to say its *success* already *greatly exceeds* my anticipations. I find my Southern friends true to their professions and will in a short time give them still greater facilities for obtaining goods promptly, and the *best articles* at the *lowest prices*. See fly leaf in front.

MANUFACTURING.—I am now largely engaged in manufacturing instruments of all kinds, chairs, cases, lathes, and machinery, also gold foil. These being made chiefly under my own supervision, I can reccommend them to the profession as of *superior qualitiy*, and at lower prices than such goods are usually sold.

VULCANITE WORK.—I am as heretofore prepared to supply the profession with everything in this line.

Furnishing the gum, packing, and vulcanizing,

| | |
|---|---|
| Common Gum, each piece, ........................ | $3 00 |
| Superfine " " " ........................ | 5 00 |
| Polishing, Extra, ................................ | 2 00 |
| Polishing Amber Base, ............................ | 3 00 |

